# Kallikrein 6 as a Serum Prognostic Marker in Patients with Aneurysmal Subarachnoid Hemorrhage

**DOI:** 10.1371/journal.pone.0045676

**Published:** 2012-09-25

**Authors:** Eduardo Martínez-Morillo, Anastasia Diamandis, Alexander D. Romaschin, Eleftherios P. Diamandis

**Affiliations:** 1 Samuel Lunenfeld Research Institute, Joseph and Wolf Lebovic Health Centre, Mount Sinai Hospital, Toronto, Ontario, Canada; 2 Department of Clinical Biochemistry, Toronto General Hospital, University Health Network, Toronto, Ontario, Canada; 3 Li Ka Shing Knowledge Institute, St. Michael’s Hospital, Department of Laboratory Medicine and Pathobiology, University of Toronto, Toronto, Ontario, Canada; 4 Department of Laboratory Medicine and Pathobiology, University of Toronto, Toronto, Ontario, Canada; 5 Department of Pathology and Laboratory Medicine, Mount Sinai Hospital, Toronto, Ontario, Canada; St Michael’s Hospital, University of Toronto, Canada

## Abstract

**Background:**

Aneurysmal subarachnoid hemorrhage (aSAH) is a devastating condition that frequently causes death or significant disabilities. Blood tests to predict possible early complications could be very useful aids for therapy. The aim of this study was to analyze serum levels of kallikrein 6 (KLK6) in individuals with aSAH to determine the relevance of this protease with the outcome of these patients.

**Methodology/Principal Findings:**

A reference interval for KLK6 was established by using serum samples (n = 136) from an adult population. Additionally, serum samples (n = 326) from patients with aSAH (n = 13) were collected for 5 to 14 days, to study the concentration of KLK6 in this disease. The correlation between KLK6 and S100B, an existing brain damage biomarker, was analyzed in 8 of 13 patients. The reference interval for KLK6 was established to be 1.04 to 3.93 ng/mL. The mean levels in patients with aSAH within the first 56 hours ranged from 0.27 to 1.44 ng/mL, with lowest levels found in patients with worse outcome. There were significant differences between patients with good recovery or moderate disability (n = 8) and patients with severe disability or death (n = 5) (mean values of 1.03 ng/mL versus 0.47 ng/mL, respectively) (p<0.01). There was no significant correlation between KLK6 and S100B.

**Conclusions/Significance:**

Decreased serum concentrations of KLK6 are found in patients with aSAH, with the lowest levels in patients who died.

## Introduction

Non-traumatic subarachnoid hemorrhage (SAH), a form of hemorrhagic stroke characterized by the accumulation of blood in the subarachnoid space surrounding the brain, represents approximately 5% of all strokes and it is a devastating condition that frequently causes death or significant disabilities in affected patients [1]–[2]. Ruptured intracranial aneurysms are responsible for spontaneous SAH in 85% of cases, affecting up to 30,000 individuals per year in the United States [3]–[4]. Although case fatality rate after aneurysmal SAH (aSAH) has decreased in the last decades, about 40–50% of patients still die [5]–[6]. Besides, 20–30% of the survivors are left with disabilities and less than 1/3 of patients regain their previous occupation and life style [7].

Diagnostic methods currently used are computed tomography (CT) scanning of brain and lumbar puncture for analysis of cerebrospinal fluid when the CT scan is negative [1]. However, SAH is a medical emergency that is misdiagnosed in 25 to 50% of patients on their first physician consultation [8]. Moreover, in patients with aSAH who survive the first hours after hemorrhage, three main neurological complications can appear: rebleeding, delayed brain ischemia by a cerebral vasospasm and hydrocephalus [3].

Neurosin or kallikrein-related peptidase 6 (KLK6) is a protease encoded by the KLK6 gene, localized on chromosome 19q13.4 [9]. This protein is expressed in many tissues but its expression is highest in brain and spinal cord [10]. Although the physiological functions of KLK6 in the brain and spinal cord are poorly understood, several studies have linked this protein with glioma [11], Alzheimer’s disease [12]–[13], vascular dementia [12], [14] and spinal cord injury [15]–[16]. Several authors have shown that this protein is mainly expressed by oligodendrocytes and it may play an important role in remyelination after insult to the central nervous system [16]–[19]. We hypothesize that the secretion of KLK6 and subsequently, the blood concentration of this protein could be altered after brain injury as a result of its involvement in a repair mechanism of central nervous system and therefore, the aim of this study was to analyze serum levels of KLK6 in individuals with aSAH to determine the relevance of this protease with the outcome of these patients.

Serum levels of KLK6 were measured in serum samples from an adult population (to establish reference values) and in serial samples from patients with aSAH. The association between S100B protein and KLK6 was also studied.

## Results

### Reference Interval for KLK6 in Adults

The reference interval for KLK6 was established to be 1.04 (90%CI: 0.86–1.21) to 3.93 ng/mL (90%CI: 3.75–4.11). There were no significant differences with gender and there was a weak but highly significant positive correlation with age (Spearman rho = 0.205, p<0.0001). The reference change value (RCV) bi-directional (95% confidence) calculated for KLK6 was 35% [20].

### KLK6 Levels in Patients with SAH

**Figure 1:**
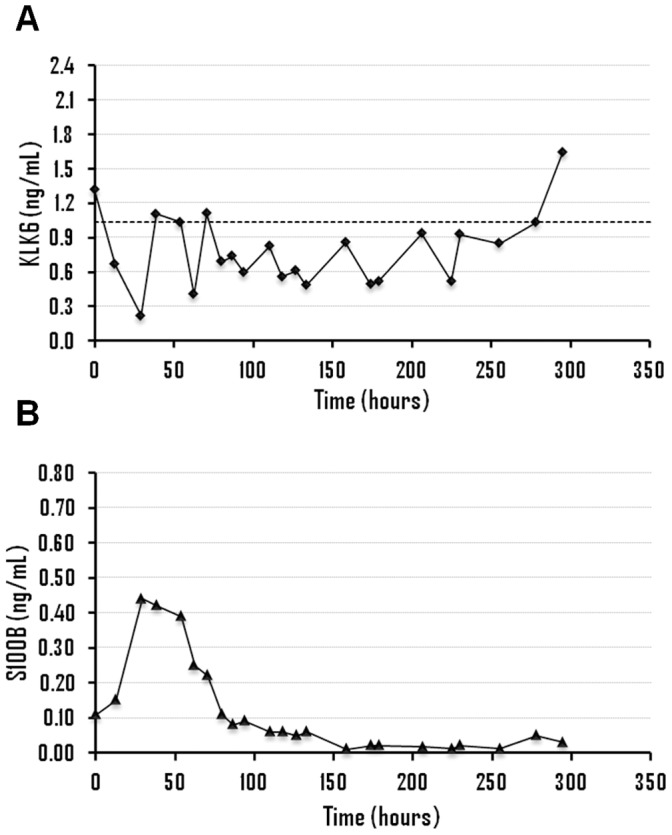
Time evolution of kallikrein 6 (A) and S100B levels (B), in ng/mL, for patient 12. The dashed line represents the lower reference limit of kallikrein 6 (1.04 ng/mL).

**Figure 2:**
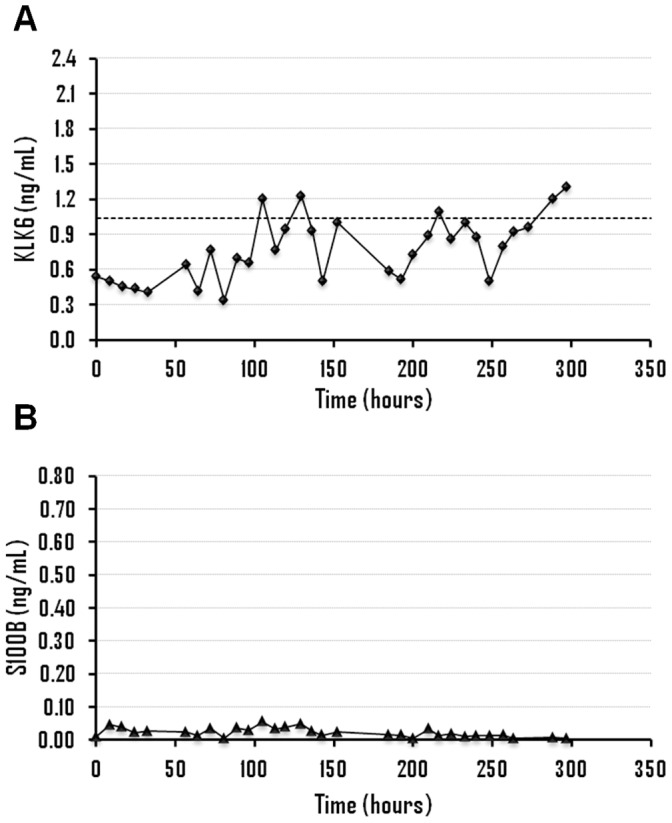
Time evolution of kallikrein 6 (A) and S100B levels (B), in ng/mL, for patient 8.

**Figure 3:**
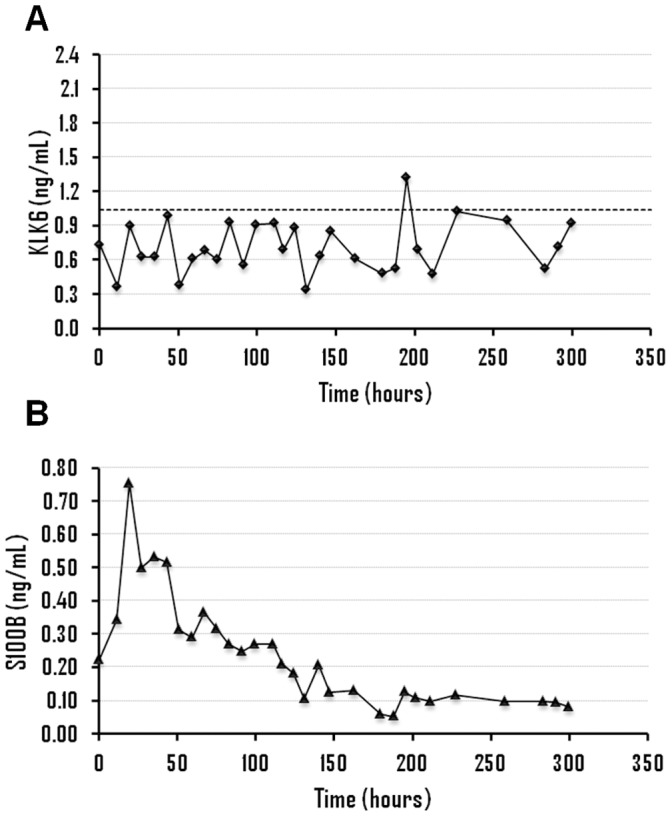
Time evolution of kallikrein 6 (A) and S100B levels (B), in ng/mL, for patient 6.

**Figure 4:**
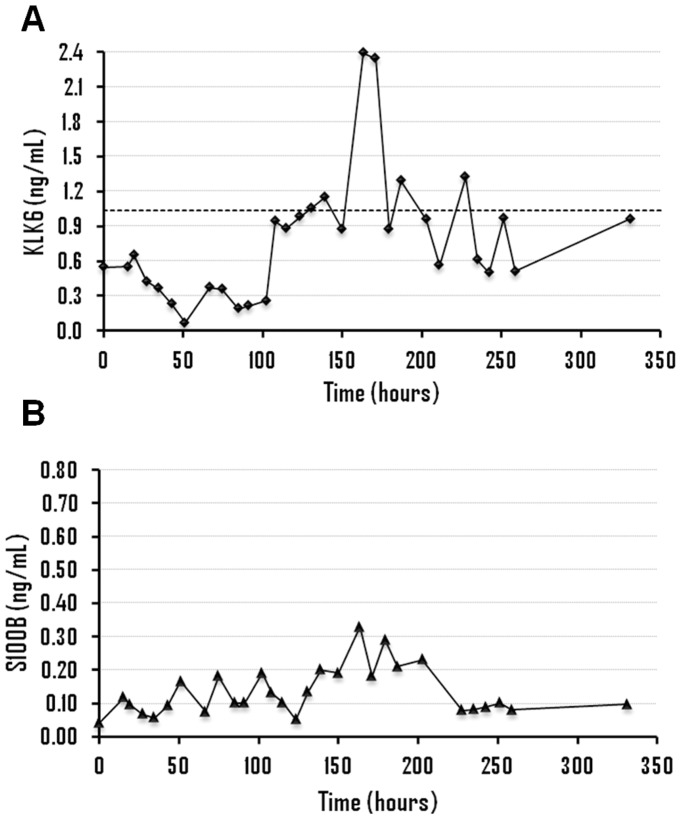
Time evolution of kallikrein 6 (A) and S100B levels (B), in ng/mL, for patient 2.

**Figure 5:**
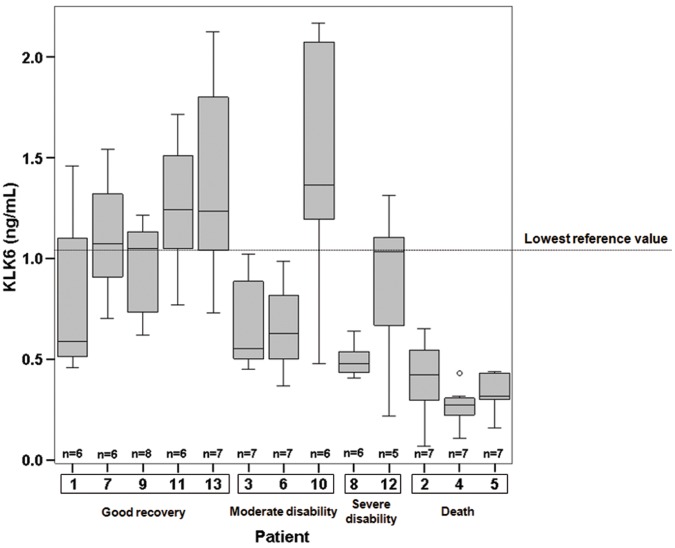
Box and Whisker diagram of serum kallikrein 6 levels (ng/mL) in patients with subarachnoid hemorrhage (n = 13), within the first 56 hours. The number of samples for each patient varies from 5 to 8.

The clinical information of patients with aSAH as well as the results obtained for KLK6 and S100B for all samples is shown in Figures 1, 2, 3 and 4 and Supporting Information S1. All patients (n = 13) displayed KLK6 concentrations below the lower reference limit, at least in some of the samples. Most informative were KLK6 results in the first serum sample and in samples collected within the first 56 hours (Figure 5). Patients who died (patients 2, 4 and 5) had the lowest KLK6 levels, with initial concentrations of 0.54, 0.23 and 0.30 ng/mL and means of 0.40, 0.27 and 0.34 ng/mL, respectively (table 1). The two patients with severe disability (patients 8 and 12) showed initial levels of 0.54 and 1.32 ng/mL and means of 0.49 and 0.87 ng/mL, respectively. However, the KLK6 levels in patients with good recovery or moderate disability (patients 1, 3, 6, 7, 9, 10, 11 and 13) were higher, with initial concentrations ranging from 0.73 to 2.17 ng/mL and means between 0.66 and 1.44 ng/mL. The mean concentration of KLK6 in the initial sample from patients with severe disability or death (n = 5) was significantly lower than the mean of patients with good recovery or moderate disability (n = 8), 0.59 versus 1.36 ng/mL, respectively (p = 0.01). We further calculated the mean KLK6 values for each patient over the first 56 hours (Mean of individual patients, MIP). Then, we compared the means of MIPs for the two groups (severe disability or death, mean of MIPs = 0.47 ng/mL; good recovery or moderate disability, mean of MIPs = 1.03 ng/mL) (p<0.01). The mean of MIPs in patients with worse outcome (0.47 ng/mL) was also significantly lower than the lowest reference limit of 1.04 ng/mL (p = 0.01).

**Table 1:** Serum concentrations of KLK6 and S100B (ng/mL) in the initial sample and all samples collected within the first 56 hours from patients with subarachnoid hemorrhage.

PatientNo.	KLK6(initial)	FC*	KLK6(56 hours)†	FC*	S100B (initial)	S100B(56 hours)†	Outcome§
**1**	1.46	+1.4	0.78±0.40	−1.3			GR
**7**	1.54	+1.5	1.10±0.30	+1.1			GR
**9**	1.22	+1.2	0.96±0.23	−1.1	0.08	0.07±0.02	GR
**11**	1.05	+1.0	1.25±0.34	+1.2			GR
**13**	1.72	+1.7	1.40±0.52	+1.3			GR
**3**	1.02	+1.0	0.69±0.24	−1.5	0.05	0.05±0.01	MD
**6**	0.73	−1.4	0.66±0.24	−1.6	0.22	0.45±0.18	MD
**10**	2.17	+2.1	1.44±0.62	+1.4			MD
**8**	0.54	−1.9	0.49±0.08	−2.1	0.01	0.03±0.01	SD
**12**	1.32	+1.3	0.87±0.43	−1.2	0.11	0.30±0.10	SD
**2**	0.54	−1.9	0.40±0.20	−2.6	0.04	0.09±0.04	D
**4**	0.23	−4.5	0.27±0.10	−3.9	0.38	0.37±0.11	D
**5**	0.30	−3.5	0.34±0.10	−3.1	0.06	0.33±0.24	D

* **FC:** Fold change with respect to lower reference limit (1.04 ng/mL).

† Values are expressed as mean ± standard deviation.

§ GR: Good recovery, MD: Moderate disability, SD: Severe disability, D: Death.

The KLK6 results obtained during the first 56 hours proved to be more informative than the results obtained in the following samples since the MIP values obtained from 56 to 127 hours were not significantly different between groups, 0.52 ng/mL (severe disability/death) versus 0.83 ng/mL (moderate disability/good recovery) (p = 0.1).

### Association between KLK6 and S100B

The association between serum levels of KLK6 and S100B (an established serum marker of brain injury) was analyzed in 8 of 13 patients with aSAH (patients 2, 3, 4, 5, 6, 8, 9 and 12), 5 women and 3 men, including patients with good recovery (n = 1), moderate disability (n = 2), severe disability (n = 2) and patients who died (n = 3). There was no significant association between the concentrations of these two proteins when the values obtained within the first 56 hours (n = 54) were analyzed with the Spearman’s correlation test. Thus, the information extracted from these two proteins about the outcome was different in some of the patients studied.

Analyzing the concentrations of KLK6 and S100B within the first 56 hours, it was observed that S100B showed a peak of concentration in patients 4, 5, 6 and 12, with mean levels ranging from 0.30 to 0.45 ng/mL (table 1). On the other hand, the lowest levels of KLK6 were observed in patients 2, 4, 5 and 8, with mean concentrations between 2.1 and 3.9-fold lower than the lowest reference limit. Based on these results, patients 4 and 5, who died from brain damage, were classified as high risk based on the concentration of both proteins and patients 3 and 9, with moderate disability and good recovery, respectively, were classified as low risk, according to the levels of KLK6 and S100B. On the other hand, patient 2 (who died) was classified as high risk only with KLK6, with a mean level 2.6-fold lower than the lowest reference limit. In patients with severe disability, the concentrations of the two proteins was discrepant since patient 8 was classified as high risk only with KLK6 (mean concentration 1.9-fold lower than the lowest reference limit) while patient 12 was classified as high risk only with S100B (mean concentration of 0.30 ng/mL). Finally, patient 6 (with moderate disability) was classified as high risk according to S100B (mean concentration of 0.45 ng/mL). Therefore, KLK6 seems to allow a better classification (based on outcome) of patients 2, 6 and 8 and S100B of patient 12.

## Discussion

To our knowledge, this is the first study that evaluates the serum levels of KLK6 in patients with SAH. KLK6 serum concentration in patients with aSAH was significantly lower than the concentration of healthy individuals. All patients studied had KLK6 serum levels below the reference limit in some, or all of the samples analyzed, with lowest concentrations in patients with worse outcome. Thus, mean levels of serum KLK6, within the first 56 hours, in patients with severe disability or death (0.47 ng/mL) were significantly lower than the lowest reference limit (1.04 ng/mL) while mean levels in patients with good recovery or moderate disability were close to normal (1.03 ng/mL). Serum samples collected within the first 48–72 hours are more informative for two reasons. First, some life-threatening complications such as delayed brain ischemia due to cerebral vasospasm rarely happen within 72 hours, occurring most frequently 7 to 10 days after SAH [1], so that the information obtained during the first 2–3 days could be very useful in aiding selection of specific treatments. Second, the peak concentration of S100B in the majority of patients occurs within 24–48 hours after SAH, without further elevations. Therefore, we selected the first 56 hours since our data reveal that KLK6 concentration in samples obtained after this time correlated less strongly with outcome.

No significant association between KLK6 and S100B was observed. Moreover, the predicted outcome according to the concentrations of these two biomarkers was different in some patients. Thus, while both markers predicted high risk in patients 4 and 5 (patients with vasospasm and brain infarction, respectively, who eventually died) and lower risk in patients 3 and 9 (patients with better outcome), the predicted outcome in patients 2, 6, 8 and 12 was different. For patient 12 (severe disability) there was an S100B concentration peak with a mean of 0.30 ng/mL. Mean concentration of KLK6 was within the reference limit but with a minimum value of 0.22 ng/mL after 29 hours, which coincides with the peak concentration observed for S100B (Figure 1). Patient 8 (severe disability) had a mean level of KLK6 almost two times lower than the lowest reference limit, but normal concentrations of S100B (Figure 2). Patient 6 (moderate disability) showed a large elevation in S100B levels with a mean concentration of 0.45 ng/mL and levels of KLK6 around 1.4 times lower than the lowest reference limit (Figure 3). Finally, patient 2 (who died) had very low levels of KLK6 with a minimum value of 0.07 ng/mL and a sustained elevation in S100B concentrations (Figure 4). The subsequent dramatic elevation of both KLK6 and S100B proteins after 170 hours (see Figure 4), may be associated with the suffered bowel obstruction. This patient died of pulmonary embolism and not from brain injury and, therefore, the cognitive deficits after SAH could not be properly assessed.

**Table 2:** Characteristics of patients with aneurysmal subarachnoid hemorrhage (n = 13).

PatientNo.	Gender	Age (years)	Serum samples*	Period (days)*	Treatment†	Outcome
1	Female	44	21	9.4	EC	Good recovery
2	Female	73	30	13.8	SC	Death
3	Male	56	26	13.5	EC	Moderate disability
4	Male	48	37	12.8	SC	Death
5	Female	50	7	2.3	SC	Death
6	Female	50	30	12.5	SC	Moderate disability
7	Female	51	33	12.6	EC	Good recovery
8	Female	63	32	12.4	EC	Severe disability
9	Male	35	24	7.7	SC	Good recovery
10	Female	35	18	7.0	EC	Moderate disability
11	Female	57	30	10.8	EC	Good recovery
12	Female	53	23	12.3	SC	Severe disability
13	Female	72	15	5.3	EC	Good recovery

* Number of serum samples and period of collection (days) for each patient.

† EC: endovascular coiling, SC: surgical clipping.

KLK6 is a serine protease highly expressed in brain and spinal cord [10]. Several studies have shown that this protein is mainly expressed by mature oligodendrocytes and oligodendrocyte progenitors [16]–[18], a subset of macroglial cells whose main function is the formation of a myelin sheath around axons in the central nervous system. Bando et al. [19] suggested that KLK6 plays a role in remyelination after insult to the central nervous system. They observed a reduction in the number of oligodendrocytes and KLK6 concentration in demyelinating conditions and an elevation to initial values after remyelination. The same group further showed that KLK6 could act as a regulator of myelin proteins. On the other hand, Terayama et al. [16] reported that the expression of KLK6 by oligodendrocyte precursors is reduced after brain injury, perhaps as a result of the migration of these cells to damaged sites, to promote rapid remyelination. Other proteins studied previously in hemorrhagic stroke, such as S100B and GFAP, are mainly expressed by astrocytes, macroglial cells that play an important role in the regulation of blood-brain barrier. After brain injury, these proteins are released from damaged brain cells through the disrupted blood-brain barrier and detected in serum [21]–[22]. In contrast, KLK6 is expressed by oligodendrocytes in normal conditions. The reduction of serum concentration of this protein after hemorrhagic stroke and subsequent brain injury could indicate oligodendroglial death and axonal demyelination-remyelination.

The results described in the present study suggest a possible utility of serum levels of KLK6 as a biomarker of poor prognosis in patients with aSAH, since the lowest levels were found in patients who died or survived but with significant neurocognitive deficits. The initial concentration after patient hospitalization (<12 hours form the event) and KLK6 levels within the 2–3 days after hemorrhage seem to be the most informative in classifying patients according to their outcome, perhaps reflecting the extent of the initial brain damage suffered. The establishment of a robust reference interval, as well as the estimation of RCV for KLK6, allowed us to evaluate with high confidence the results obtained. Thus, the RCV is the difference required for two serial results to be considered clinically different. For KLK6 this value was 35%, which means that any change in KLK6 concentration greater than 35% could be associated with a disease, with 95% confidence, and not to intraindividual normal variation. However, this study has some limitations. The number of studied patients with aSAH was low and the serum samples were collected 9 or more hours after diagnosis of the disease (for patient 1 more than 24 hours). Since most of them were referred to St. Michael’s hospital from other hospitals, samples in the first hours after hemorrhage could not be obtained. In addition, the patients were not classified according to the Glasgow Outcome Scale but using the clinical information available. Further analyses with a larger number of patients should be conducted to further elucidate the potential of this protein as a prognostic biomarker after hemorrhagic stroke, either alone, or in combination with existing markers.

## Materials and Methods

### Ethics Statement

Ethics approval for the collection of samples was obtained from the Institutional Review Board of St. Michael’s Hospital.

### KLK6 in an Adult Population

Serum samples from healthy volunteers (n = 136), 73 women and 63 men, 20 to 86 years old were collected. Additionally, serial serum samples (n = 72) from four of these individuals, 2 men and 2 women, were collected every 2–3 days for two months. Samples were stored at −80°C until analyzed. The reference interval and the RCV for KLK6 were calculated from these samples.

### Study Population with SAH

This is a retrospective study were frozen serum samples from patients with aSAH (n = 13) and admitted in St. Michael’s Hospital (Toronto), between June and October 2006, were used for the analysis of KLK6. The diagnosis of SAH was based on medical history and a positive CT scan. The aneurysm was secured with neurosurgical clipping or endovascular coiling. Serum samples were obtained as soon as possible after admission of individuals to the intensive care unit and then approximately every 9–11 hours, for 5 to 14 days. Between 7 and 37 samples for each patient with SAH were collected, with a total of 326 samples, which were stored at −80°C until analyzed. The main characteristics of the study population are shown in table 2.

### Outcome

Since it was not possible to classify the outcome of patients with aSAH according to the Glasgow Outcome Scale, they were classified into four groups according to available information in their medical records (Table S1). Thus, patients who recovered without neurocognitive deficits were classified in the group “good recovery”, patients who survived with mild neurocognitive deficits were classified as “moderate recovery”, patients who survived with significant neurocognitive deficits were classified as “severe disability” and patients who finally died were included in the group “death”.

### KLK6 and S100B Measurement

The KLK6 concentrations were analyzed in all patients and the concentrations of S100B in 8 of 13 patients (due to serum sample depletion). KLK6 was analyzed with an in-house spectrophotometric ELISA assay with detection limit of 0.03 ng/mL and dynamic range of 0.03–10 ng/mL. The within-run coefficient of variation (CV) is from 2 to 3% at levels of KLK6 of 2.1, 3.3 and 6.3 ng/mL and between-run CV is from 3 to 7% at levels of 0.6, 1.5 and 5.5 ng/mL [20]. The concentrations of S100B were measured with a fully-automated electrochemoluminometric immunoassay (Elecsys S100®; Roche Diagnostics, Penzberg, Germany), with detection limit of 0.005 ng/mL.

### Statistical Analysis

Statistical analyses were performed using SPSS software, version 15.0 (SPSS Inc., Chicago, IL). A p-value less than 0.05 was considered to be statistically significant. The distributions were analyzed using parametric or non-parametric tests. Normal distribution was evaluated using Shapiro-Wilk test and by inspection of the Q-Q plot. The reference interval and associated confidence intervals (CI) for KLK6 were calculated using the parametric procedure. The RCV was estimated according to the method of Fraser [23]. Means and standard deviations were calculated for descriptive and comparative purposes. Student’s t-tests were performed for comparison between groups and with the lower reference limit. Associations between variables were reported by Spearman’s rank correlation coefficient.

## Supporting Information

Table S1 (DOC)

Supporting Information S1 (DOC)
