# Supplementary material for: Kallikrein 6 as a Serum Prognostic Marker in Patients with Aneurysmal Subarachnoid Hemorrhage
Source: PLoS One. 2012 Sep 25;7(9):e45676. doi: 10.1371/journal.pone.0045676 (PMC3458071; doi:10.1371/journal.pone.0045676)
Supplement: Table S1 — (DOC) [file pone.0045676.s001.doc]

**Table S1─**Initial assessment of patients (WFNS scale) and established outcome according to available medical information.

| **Patient** | **WFNS*** | **Medical record** | **Outcome†** |
| --- | --- | --- | --- |
| 1 | 1 | No neurocognitive deficits | GR |
| 2 | 4 | Death | D |
| 3 | 5 | Mild neurocognitive deficits | MD |
| 4 | 5 | Death | D |
| 5 | N/A | Death | D |
| 6 | 4 | Mild neurocognitive deficits | MD |
| 7 | N/A | No neurocognitive deficits | GR |
| 8 | N/A | Significant neurocognitive deficits | SD |
| 9 | 2-3 | No neurocognitive deficits | GR |
| 10 | 1-2 | Mild neurocognitive deficits | MD |
| 11 | 1 | No neurocognitive deficits | GR |
| 12 | 4 | Significant neurocognitive deficits | SD |
| 13 | 1 | No neurocognitive deficits | GR |
| N/A: non-available  *WFNS: World Federation of Neurological Surgeons scale  †GR: Good recovery, MD: Moderate disability, SD: Severe disability, D: Death | | | |
