# Supplementary material for: Kallikrein 6 as a Serum Prognostic Marker in Patients with Aneurysmal Subarachnoid Hemorrhage
Source: PLoS One. 2012 Sep 25;7(9):e45676. doi: 10.1371/journal.pone.0045676 (PMC3458071; doi:10.1371/journal.pone.0045676)
Supplement: Supporting Information S1 — (DOC) [file pone.0045676.s002.doc]

**Patients (n=13) with aneurysmal subarachnoid hemorrhage: clinical information and KLK6/S100B results.**

- **Patient 1**

**Gender:** Female

**Age:** 44 years

**Glasgow Coma Scale (GCS):** 15

**Number of aneurysms:** 1

**Localization:** Anterior communicating artery

**Fisher computed tomography (CT) grading scale:** 3

**Treatment:** Endovascular coiling

**Complications:** No

**Outcome:** Patient recovered without major deficits

**Dates**

**First serum sample:** August 22^th^ (8 pm)

**Diagnosis of SAH:** August 20^th^

**Surgery:** August 23^th^


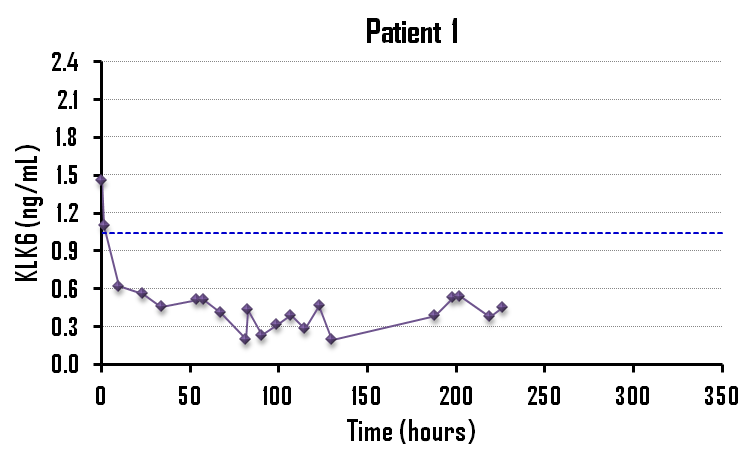


**Figure S1─**Time evolution of kallikrein 6 levels (ng/mL) in patient 1. The blue dashed line represents the lower reference limit (1.04 ng/mL).

- **Patient 2**

**Gender:** Female

**Age:** 73 years

**GCS:** 10

**Number of aneurysms:** 1

**Treatment:** Insertion of external ventricular drainage + surgical clipping

**Complications:** Bowel obstruction on October 20^th^ treated surgically

**Outcome:** Patient died 23 days after her brain injury of pulmonary embolism.

She was recovering with some cognitive deficit but not severe until death

**Dates**

**First serum sample:** October 12^th^ (11 am)

**Diagnosis of SAH:** October 11^th^

**Surgery:** October 13^th^

- **Patient 3**

**Gender:** Male

**Age:** 56 years

**GCS:** 4

**Number of aneurysms:** 1

**Treatment:** Insertion of external ventricular drainage + endovascular coiling

**Complications:** Mild hydrocephalus following surgery

**Outcome:** Patient survived with mild cognitive impairment

**Dates**

**First serum sample:** July 23^th^ (8:30 am)

**Diagnosis of SAH:** July 22^th^

**Surgery:** July 22^th^


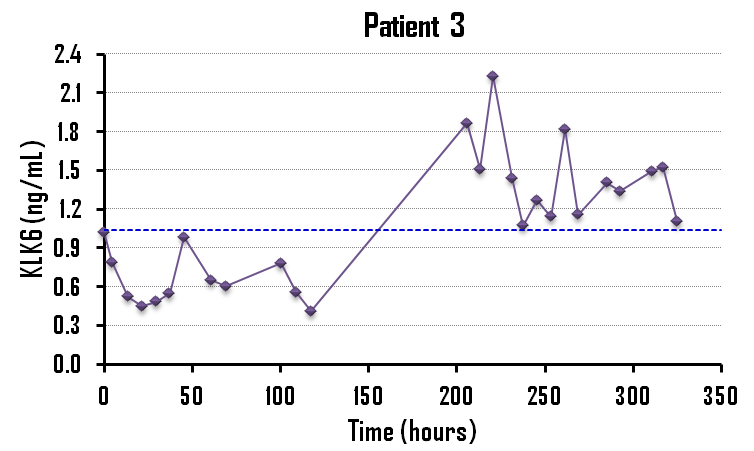


**Figure S2─**Time evolution of kallikrein 6 levels (ng/mL) in patient 3.

**
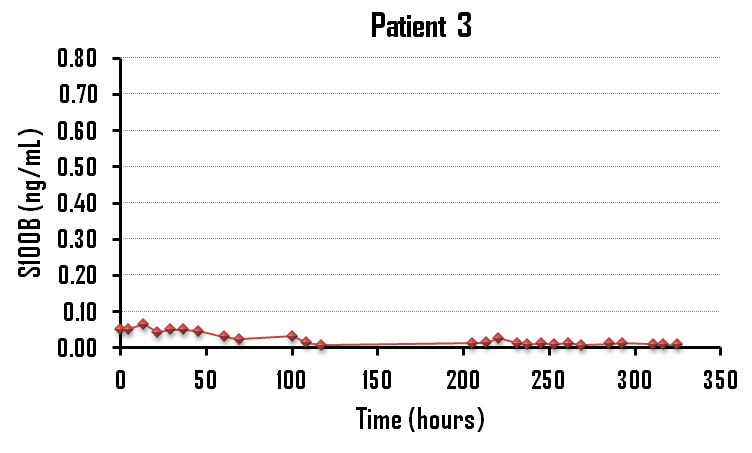
**

**Figure S3─**Time evolution of S100B levels (ng/mL) in patient 3.

- **Patient 4**

**Gender:** Male

**Age:** 48 years

**GCS:** 4

**Hunt-Hess grade:** 5

**Number of aneurysms:** 1

**Localization:** Anterior communicating artery

**Fisher CT grading scale:** 3

**Treatment:** Surgical evacuation of large clot and insertion of external ventricular drainage + surgical clipping

**Complications:** **Vasospasm** and increased persistent intracranial pressure

**Outcome:** Patient died of complications of aneurysm rupture on June 25^th^

**Dates**

**First serum sample:** June 12^th^ (8:30 am)

**Diagnosis of SAH:** June 11^th^

**Surgery:** June 11^th^


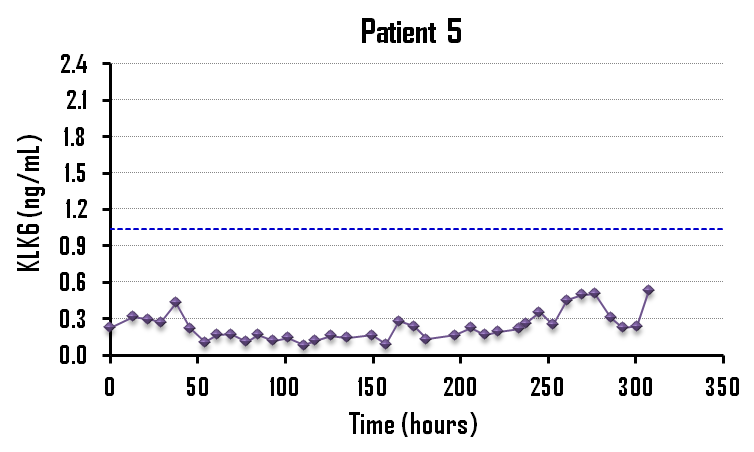


**Figure S4─**Time evolution of kallikrein 6 levels (ng/mL) in patient 4.


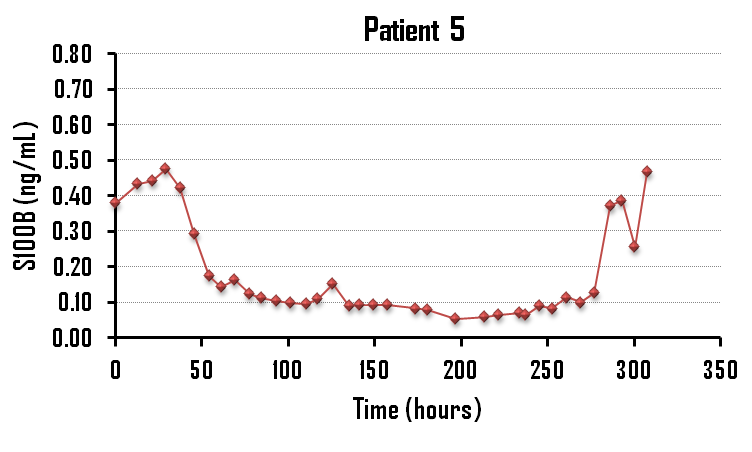


**Figure S5─**Time evolution of S100B levels (ng/mL) in patient 4.

- **Patient 5**

**Gender:** Female

**Age:** 50 years

**Number of aneurysms:** 1

**Localization:** Middle cerebral artery

**Treatment:** Surgical clipping

**Complications:** Malignant cerebral edema and **brain infarction** on June 20^th^

**Outcome:** Patient died on June 20^th^ due to brain stem compression and loss of autonomic function

**Dates**

**First serum sample:** June 17^th^ (11:30 pm)

**Diagnosis of SAH:** June 17^th^

**Surgery:** June 17^th^


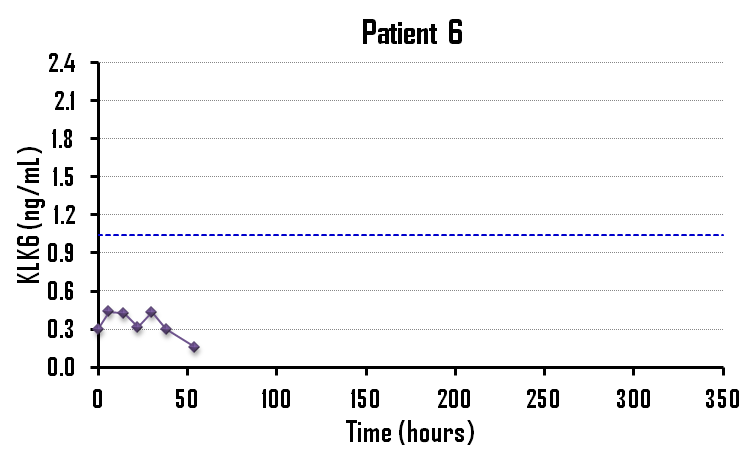


**Figure S6─**Time evolution of kallikrein 6 levels (ng/mL) in patient 5.


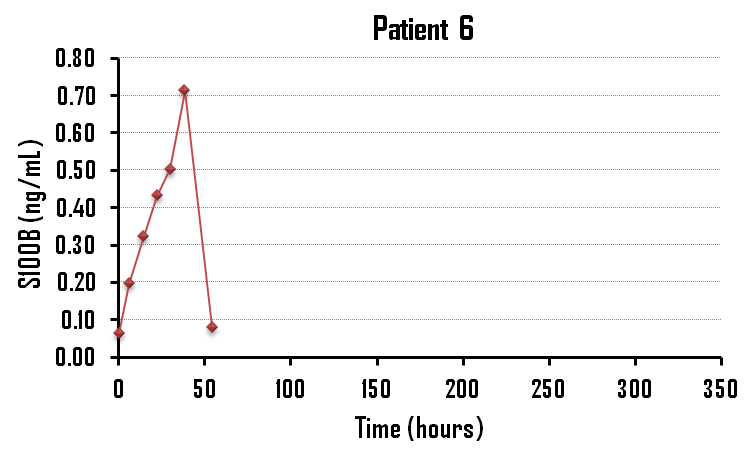


**Figure S7─**Time evolution of S100B levels (ng/mL) in patient 5.

- **Patient 6**

**Gender:** Female

**Age:** 50 years

**GCS:** 10

**Number of aneurysms:** 1

**Localization:** Pericallosal artery

**Treatment:** Surgical clipping and evacuation of large hematoma with suction

**Complications:** Hydrocephalus on October 13^th^

**Outcome:** Patient survived with moderate cognitive deficit and some recurring seizures

**Dates**

**First serum sample:** October 11^th^ (6:30 pm)

**Diagnosis of SAH:** October 11^th^

**Surgery:** October 11^th^

- **Patient 7**

**Gender:** Female

**Age:** 51 years

**Number of aneurysms:** 1

**Treatment:** Endovascular coiling

**Complications:** No

**Outcome:** Patient survived with no cognitive deficits or any major neurological manifestations

**Dates**

**First serum sample:** July 13^th^ (10:30 pm)

**Diagnosis of SAH:** July 13^th^

**Surgery:** July 13^th^


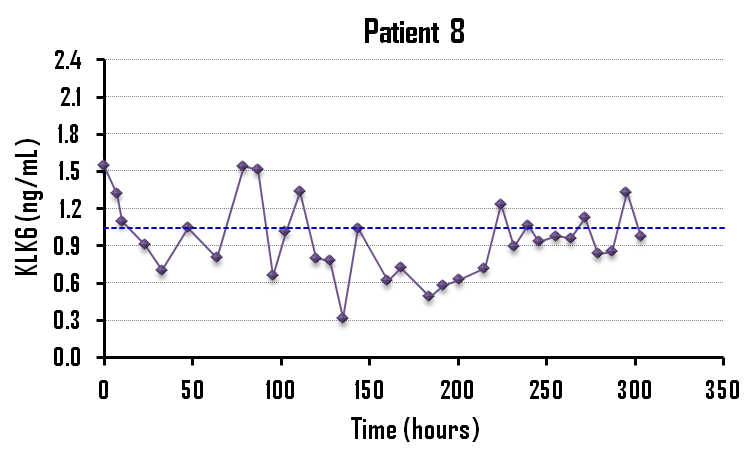


**Figure S8─**Time evolution of kallikrein 6 levels (ng/mL) in patient 7.

- **Patient 8**

**Gender:** Female

**Age:** 63 years

**Number of aneurysms:** 1

**Treatment:** Endovascular coiling and insertion of transfrontal extraventricular shunt to relieve intracranial pressure

**Complications:** No

**Outcome:** Patient survived but with poor cognitive functions and had to be institutionalized for continuous care

**Dates**

**First serum sample:** October 12^th^ (5:30 am)

**Diagnosis of SAH:** October 11^th^

**Surgery:** October 11^th^

- **Patient 9**

**Gender:** Male

**Age:** 35 years

**GCS:** 14

**Number of aneurysms:** 1

**Localization:** Anterior communicating artery

**Fisher CT grading scale:** 3

**Treatment:** Surgical clippling

**Complications:** No

**Outcome:** Patient recovered with no deficits

**Dates**

**First serum sample:** October 10^th^ (2:30 am)

**Diagnosis of SAH:** October 9^th^

**Surgery:** October 9^th^


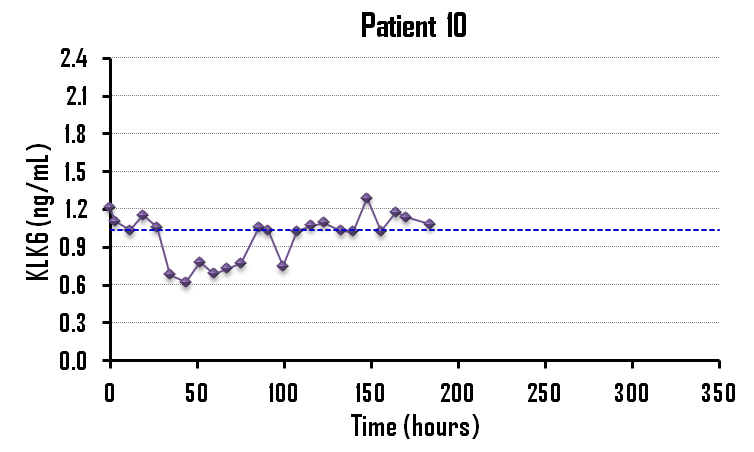


**Figure S9─**Time evolution of kallikrein 6 levels (ng/mL) in patient 9.

**
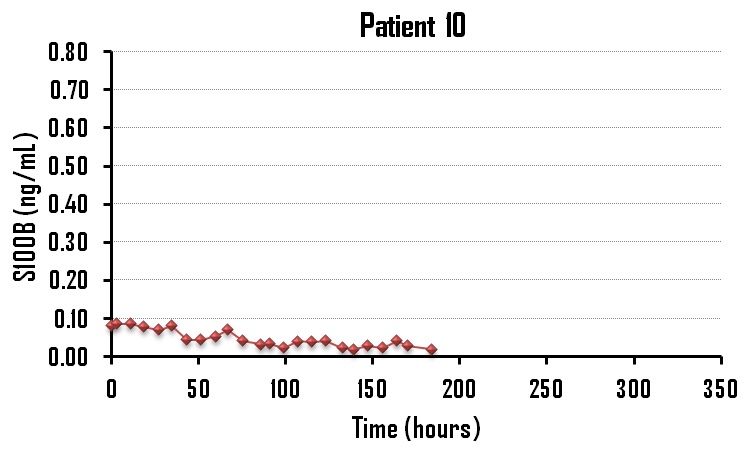
**

**Figure S10─**Time evolution of S100B levels (ng/mL) in patient 9.

- **Patient 10**

**Gender:** Female

**Age:** 35 years

**Number of aneurysms:** Multiple

**Treatment:** Endovascular coiling

**Complications:** No

**Outcome:** Patient recovered with mild memory deficit

**Dates**

**First serum sample:** September 11^th^ (6 am)

**Diagnosis of SAH:** September 10^th^

**Surgery:** September 12^th^


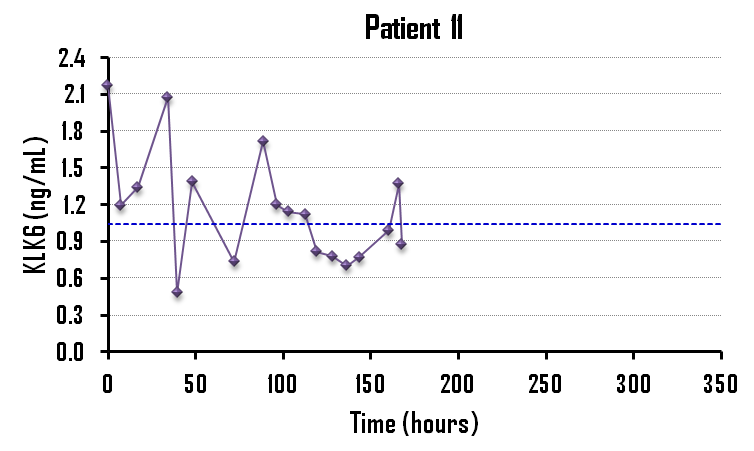


**Figure S11─**Time evolution of kallikrein 6 levels (ng/mL) in patient 10.

- **Patient 11**

**Gender:** Female

**Age:** 57 years

**GCS:** 15

**Number of aneurysms:** 1

**Treatment:** Endovascular coiling

**Complications:** No

**Outcome:** Patient recovered with no deficits

**Dates**

**First serum sample:** July 12^th^ (10:30 am)

**Diagnosis of SAH:** July 11^th^

**Surgery:** July12^th^


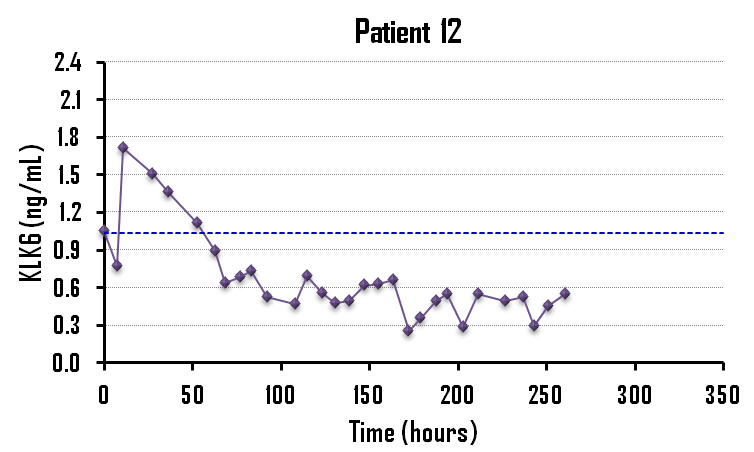


**Figure S12─**Time evolution of kallikrein 6 levels (ng/mL) in patient 11.

- **Patient 12**

**Gender:** Female

**Age:** 53 years

**GCS:** 7

**Number of aneurysms:** 1

**Treatment:** Surgical clipping

**Complications:** No

**Outcome:** Patient survived but has neurological symptoms including limping during walking, headaches, chest pain, back pain, toe cramps, lack of co-ordination on left side. She has problems with concentration, memory lapse, anxiety and depression

**Dates**

**First serum sample:** October17^th^ (3:30 pm)

**Diagnosis of SAH:** October 16^th^

**Surgery:** October 18^th^

- **Patient 13**

**Gender:** Female

**Age:** 72 years

**GCS:** 15

**Number of aneurysms:** 1

**Treatment:** Endovascular coiling

**Complications:** No

**Outcome:** Patient recovered with no deficits

**Dates**

**First serum sample:** July 14^th^ (9:30 pm)

**Diagnosis of SAH:** July 14^th^

**Surgery:** July 15^th^


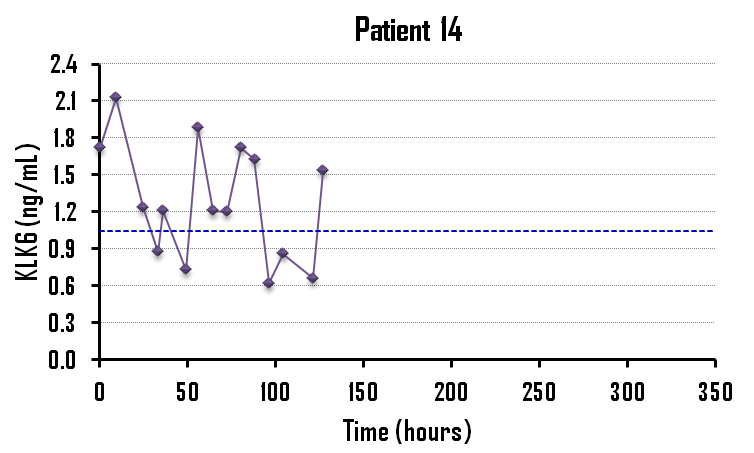


**Figure S13─**Time evolution of kallikrein 6 levels (ng/mL) in patient 13.
